# Supplementary material for: First Experimental Study of the Influence of Extracellular Vesicles Derived from Multipotent Stromal Cells on Osseointegration of Dental Implants
Source: Int J Mol Sci. 2021 Aug 16;22(16):8774. doi: 10.3390/ijms22168774 (PMC8395855; doi:10.3390/ijms22168774)
Supplement: Supplementary file 1 [file ijms-22-08774-s001.zip › ijms-1318518-supplementary.pdf]

Supplementary Materials:

First Experimental Study Of The Influence Of Extracellular Vesicles Derived From Multipotent Stromal Cells On Osseointegration Of Dental Implants

Igor Maiborodin [1, 2,*], Aleksandr Shevela [1, 3], Vera Matveeva [1], Vitaly Morozov [1], Michael Toder [3], Sergey Krasil'nikov [1], Alina Koryakina [3], Andrew Shevela [1] and Oleg Yanushevich [4]

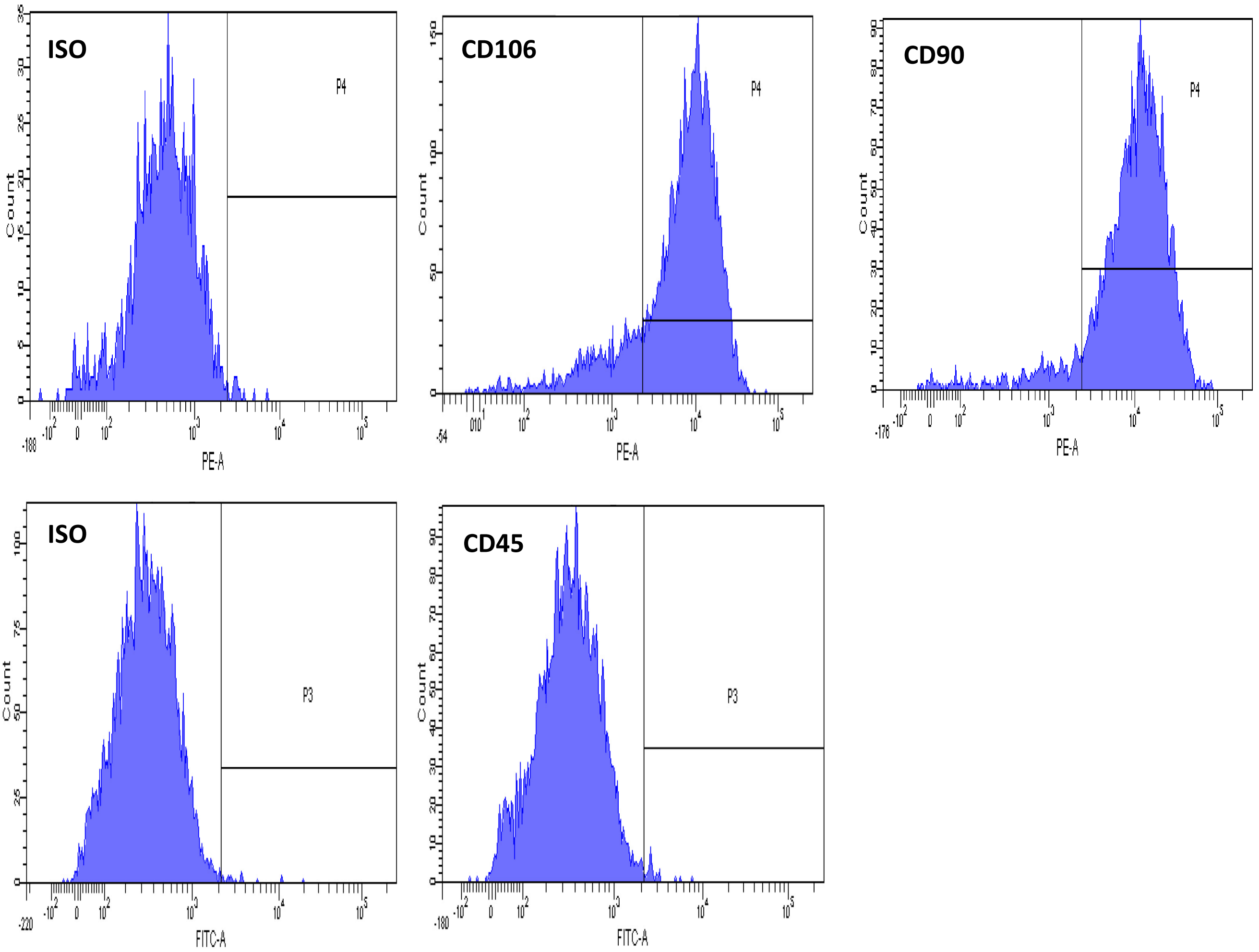

Figure S1. The phenotype of rat mesenchymal stem cells (MSCs) derived from the bone-marrow. Data indicate flow cytometry analysis. The MSC of rat were stained with specific antibodies or isotype controls. The samples were analyzed on a flow cytometer "FACSAria" III using FACSDiVa Version 6.1.3. software (Becton Dickinson).
